# Remote Work Decreases Psychological and Physical Stress Responses, but Full-Remote Work Increases Presenteeism

**DOI:** 10.3389/fpsyg.2021.730969

**Published:** 2021-09-30

**Authors:** Akiyoshi Shimura, Katsunori Yokoi, Yoshiki Ishibashi, Yusaku Akatsuka, Takeshi Inoue

**Affiliations:** ^1^Department of Psychiatry, Tokyo Medical University, Tokyo, Japan; ^2^Department of Research and Development, Children and Future Co., Ltd., Tokyo, Japan; ^3^School of Medicine, International University of Health and Welfare, Chiba, Japan; ^4^Department of Preventive Medicine and Public Health, Keio University, Tokyo, Japan

**Keywords:** occupational & industrial medicine, job stress, remote work, occupational mental health, presenteeism, COVID-19

## Abstract

**Introduction:** Remote work was widely promoted in 2020, as a result of the COVID-19 pandemic. However, the effects of remote work on psychological and physical stress responses and presenteeism of workers remain unclear. This research aims to provide empirical evidence of the implications for people and organizations of this new scenario of working from home.

**Methods:** A two-wave panel survey of before and after the pandemic was performed to investigate the effects of remote work on these aspects among office workers. A total of 3,123 office workers from 23 tertiary industries responded to a questionnaire. Participants were surveyed about their job stress conditions and sleep practices in both 2019 and 2020, who had not done remote work as of 2019 were included in the study. The effects of remote work on psychological and physical stress responses and presenteeism were analyzed by multivariate analysis, with the adjustment of age, gender, overtime, job stressors, social support, and sleep status.

**Results:** The multivariate logistic regression analysis demonstrated that remote work was associated with the reduction of psychological and physical stress responses independently of changes of job stressors, social support, sleep disturbance, and total sleep time on workdays. On the other hand, remote work of 5 days a week (full-remote) was associated with the reduction of work productivity.

**Conclusion:** Promoting remote work can reduce psychological and physical stress responses, however, full-remote work has the risk of worsening presenteeism. From the viewpoint of mental health, the review of working styles is expected to have positive effects, even after the end of the COVID-19 pandemic.

## Introduction

COVID-19 has been continuing to spread across the world, with more than 170 million confirmed cases worldwide, and more than three million deaths as of June 2021. The “Stay at home” policy has been promoted to control and mitigate the pandemic, which would reduce the burden on national healthcare systems and entire economies (Anderson et al., 2020). Working from home, also known as remote work, telework, or mobile work, is expected to reduce the risk of COVID-19 infection (Di Domenico et al., 2020; Kawashima et al., 2020), and has been widely implemented as part of the “Stay at home” policy. In fact, in the US, 35.2% of its workforce worked entirely from home in May 2020, up from 8.2% in February (Saltiel, 2020). Also, remote work became more common in Europe (Eurofound, 2020) and in Japan, the rate of implementation of remote work increased from 10% in March to 17% in June 2020 (Okubo, 2020).

The benefits of remote work remain controversial. Remote work enables a better balance of home and work life, increased flexibility and autonomy, reduction in commuting time, increased productivity, and higher morale and job satisfaction (Tavares, 2017). A meta-analysis found that there is a small positive association between remote work and organizational outcomes, such as increased productivity, employee retention, and organizational commitment (Martin and MacDonnell, 2012). On the other hand, remote work can result in social isolation and marginalization, which increases the stress of workers (Di Martino and Wirth, 1990), and a literature review reported that there was little clear evidence that remote work increases job satisfaction and productivity (Bailey and Kurland, 2002). Moreover, a research group in Europe also concluded that working from home was associated with work productivity loss caused by sickness (Steidelmüller et al., 2020), which is also known as presenteeism (Aronsson et al., 2000).

Presenteeism is particularly a concern in the fields of economics and public health, and has a greater cost than that of treatments for physical and mental illness (Loeppke et al., 2009) or absenteeism (Burton et al., 2004). Furthermore, before the COVID-19 pandemic, remote work, working from home, and telecommuting are options that some companies have been offering for the advantages described above, and eligible workers could choose their workstyle by themselves (Lapierre et al., 2016). At present, with the COVID-19 pandemic, this remote work practice has become more widespread owing to company and government regulations aiming at social distancing, and has been associated with negative effects on stress levels, mental health, and health behaviors (Czeisler et al., 2020), such as substance use (Pfefferbaum and North, 2020). As the context of remote work has changed between before and after the start of the pandemic, reexamination of the effects of remote work on mental health is required.

Not only the effects of the remote work during COVID-19 pandemic, but also the condition of the workplace is a crucial factor associated with mental health and presenteeism in the workplace. A systematic review has supported the proposition that work can be beneficial for an employee's mental health, particularly if good-quality supervision is provided and workplace conditions are favorable (Modini et al., 2016). Additionally, as another important personal factor, sleep status is strongly associated with psychological and physical stress reactions (Åkerstedt et al., 2002; Miyama et al., 2020) and presenteeism (Furuichi et al., 2020; Ishibashi and Shimura, 2020) in the workplace, and may be affected by the COVID-19 pandemic (Rajkumar, 2020; Wang et al., 2020). Some studies have assessed the associations between the COVID-19 pandemic and mental health, the working environment (Galanti et al., 2021), and home conflicts (Freisthler et al., 2021), and the advantages and disadvantages of remote work, and one study reported the positive effects of remote work on stress reactions during the pandemic (Darouei and Pluut, 2021).

To date and as far as we know, there is no single study that has analyzed the differences in the effects of remote work on stress reactions and presenteeism between before and after the start of the pandemic, and moreover, how job stressors and work environments should be adjusted. We hypothesized that remote work itself is beneficial for workers' mental health, and the controversial results of the previous studies were owing to the lack of adjustment of important confounders, such as job stressors, social support, and personal factors, such as sleep. Therefore, to analyze the effects of remote work on stress reactions and presenteeism, we performed a two-wave study of before and during the COVID-19 pandemic, analyzing job stressors, social support, and sleep status.

## Subjects and Methods

### Participants and Ethical Considerations

In 2019, before the COVID-19 pandemic, 40 companies comprising 6,855 workers participated in a survey program and agreed to the academic use of their data. The participants were recruited during the annual mental health checkup program. In 2020, 17 companies with a total of 2,336 participants withdrew from the survey program. The remaining 23 companies, which were tertiary industries located in Japan, in the areas of information technology, finance, broadcasting, music, consulting, public office, chemical industry, healthcare, fashion, printing, movie, trading, restaurant, travel agency, patent agency, and temp agency, remained in the survey. Of the remaining participants, 3,359 provided valid answers to the same questionnaire again, whereas 967 participants had missing answers, and 193 participants gave invalid answers. Among the participants who provided valid answers, the data of 3,123 participants who had never engaged in remote work in 2019 were analyzed.

This study was performed in accordance with the Helsinki Declaration, and was approved by the Tokyo Medical University Medical Ethics Review Board (study approval no.: SH3652). All the participants provided informed consent online, and data were completely anonymized.

### Measurements

We asked first participants for their demographic characteristics (age and gender), as well as their working and time conditions (frequency of the remote work performed per week, average overtime worked per month; the total additional work time that exceeded 40 h a week).

Secondly, we used validated scales to examine job environment, psychological and physical stress responses, social support, sleep status, and presenteeism as below.

#### Brief Job Stress Questionnaire

To evaluate job stressors and stress responses, the BJSQ (Shimomitsu, 2000; Ando et al., 2015) was used. The BJSQ is a 57-item self-reported Likert scale questionnaire that measures job stressors (Area A), psychological and physical stress responses presented as psychosomatic symptoms (Area B), and social support (Area C). In Area A, there are 17 items asking about job stressors consisted of quantitative job overload, qualitative job overload, physical demands, job control, skill utilization, interpersonal conflict, poor physical environment, job suitability, and meaningfulness of work (item example: “I have an extremely large amount of work to do”). In Area B, there are 29 items to evaluate psychological and physical stress responses appeared as psychosomatic symptoms consisted of vigor, irritability, fatigue, anxiety, depression, and physical complaints (item example: “I have been inwardly annoyed or aggravated”). In Area C, there are 9 items asking about social support consisted of support from the supervisor, support from colleagues, and support from family members and friends (item example: “How reliable are the co-workers when you are troubled?”). A higher score in the BJSQ indicates a more stressful job environment (Area A), severer psychological and physical stress response (Area B), and less social support (Area C). The rest 2 items are additional questions which asked the work and life satisfactions and are not used in the calculated score.

#### Pittsburgh Sleep Quality Index and Sleep Schedules

The PSQI (Buysse et al., 1989; Doi et al., 1998) was used for assessing sleep disturbance and their sleep schedules. The PSQI is a self-reported questionnaire consisting of 18 standardized questions asking the past month sleep status, and has the following seven components: C1, sleep quality; C2, sleep latency; C3, sleep duration; C4, habitual sleep efficiency; C5, frequency of sleep disturbance; C6, use of sleep medication; and C7, daytime dysfunction. C1, C6, and C7 are Likert scales [item example: “How would you rate your sleep quality overall? (C1),” “How often have you had trouble staying awake while driving, eating meals, or engaging in social activity? (C7)”], C5 is calculated from the sum of 9 subcomponents, which are Likert scales [item example: “How often have you had trouble sleeping because you wake up in the middle of the night or early morning? (C5b)”], and C2, C3, and C4 are calculated from habitual sleep schedules [item example: “When have you usually gotten up in the morning?”]. A higher score of each component and total score (global score) indicates severer sleep disturbance.

#### Work Limitations Questionnaire

To measure presenteeism, the short form of the WLQ (Lerner et al., 2001; Takegami et al., 2014) was used. Among the methods of measuring presenteeism, WLQ has the most reliable correlation with actual variations in work performance (Gardner et al., 2016). The short form of the WLQ consists of four components, i.e., physical demands, time management, mental-interpersonal demands, and output demands. Each component of the short form of the WLQ consists of 2 Likert scale questions. In this study, the WLQ %productivity loss score was used as an index of presenteeism. Item example: “Sit, stand or stay in one position for longer than 15 min while working: able all of the time.” The WLQ %productivity loss score is calculated by those answers and interpreted as the percentage of productivity loss in the past 2 weeks due to presenteeism relative to a healthy benchmark sample.

### Statistical Analysis

First, to analyze baseline differences and to clarify 1 year changes, one-way ANOVA was performed with groups categorized by frequency of remote work. Then, logistic regression analysis was performed setting the worsening of psychological and physical stress responses and presenteeism as dependent variables, and the status of remote work and adjusting factors, such as age, gender, overtime work, job stressors, social support, and sleep status as the independent variables. Worsening of psychological and physical stress responses was defined as an increase in the score of BJSQ area B, and worsening of presenteeism was defined as the increase in the WLQ %productivity loss score. A *p* < 0.05 was considered to indicate a statistically significant difference between groups. Statistical analysis was performed using IBM SPSS Statistics ver. 26 software.

## Results

Of the 3,123 participants (1,773 males and 1,350 females; mean age: 37.3 ± 10.9 years), 1,440 participants (46.1%) had not engaged in remote work in 2020. Among the other participants, 713 people (22.8%) had engaged in 1 or 2 days a week of remote work, 728 people (23.3%) had engaged in 3 or 4 days a week of remote work, and 242 (7.7%) people had engaged in 5 days a week of remote work, referred to as “full-remote” (Table 1). Dropped-out (*N* = 967/4,519; 21.4%) included various reasons, such as simply not answering the questionnaire again, job retirement, miswriting of their anonymized id, or withdrawal of the agreement of data use or informed consent. A comparison between participants who were followed up and those who dropped out is shown in Table 2. Slight but statistically significant differences were detected in several baseline variables between the participants who were followed up and those who dropped out. The correlation matrix and Cronbach α of each questionnaire are shown in Table 3.

**Table 1:** Study sample and correlations with the variables.

		**Frequency of**	**Psychological and physical**	**Presenteeism (2019) %**
		**remote work (2020)**	**stress response (2019)**	
	***N* (%)**	**Mean (SD)**	**Mean (SD)**	**Mean (SD)**
**Remote work in 2020**				
Total sample	3,123 (100%)	1.56 (1.77)	57.6 (13.7)	6.09 (4.53)
0 days/week (None)	1,440 (46.1%)	0 (0.00)	57.69 (13.49)	6.20 (4.64)
1–2 days/week	713 (22.8%)	1.48 (0.50)	57.31 (13.96)	5.80 (4.32)
3–4 days/week	728 (23.3%)	3.60 (0.49)	57.35 (13.73)	6.11 (4.39)
5 days/week (“full-remote”)	242 (7.7%)	5.00 (0.00)	57.96 (14.04)	6.27 (4.81)
**Demographics**				
Male	3,461 (61.4%)	1.58 (1.78)	55.8 (13.5)	6.13 (4.63)
Female	2,171 (38.5%)	1.54 (1.75)	59.8 (13.6)	6.05 (4.39)
	**Mean (SD)**	**Pearson's r correlation with**		
Age (years)	37.3 (10.9)	−0.147^**^	−0.095^**^	−0.152^**^
**Job status (baseline: 2019)**				
Overtime work (hours/month)	22.2 (27.0)	−0.099^**^	0.077^**^	0.093^**^
Job stressors	40.3 (6.7)	−0.208^**^	0.536^**^	0.405^**^
Social support	19.6 (5.4)	−0.017	0.359^**^	0.259^**^
**Outcomes (baseline: 2019)**				
Psychological and physical stress response	57.5 (13.7)	0.002	–	0.537^**^
Presenteeism (productivity loss) (%)	6.09 (4.53)	0.000	0.537^**^	–
**Sleep (baseline: 2019)**				
Sleep disturbance	6.45 (2.75)	0.007	0.562^**^	0.387^**^
C1: Sleep quality	1.44 (0.74)	0.042^*^	0.485^**^	0.254^**^
C2: Sleep latency	1.11 (0.98)	0.046^**^	0.316^**^	0.193^**^
C3: Sleep duration	1.54 (0.82)	−0.053^**^	0.186^**^	0.112^**^
C4: Habitual sleep efficiency	0.20 (0.57)	−0.024	0.147^**^	0.125^**^
C5: Frequency of sleep disturbance	1.03 (0.52)	−0.016	0.362^**^	0.208^**^
C6: Use of sleep medication	0.11 (0.49)	0.010	0.153^**^	0.085^**^
C7: Daytime dysfunction	1.02 (0.77)	0.005	0.483^**^	0.478^**^
Total sleep time on workdays (hours)	6.10 (1.11)	0.045^*^	−0.196^**^	−0.134^**^
Total sleep time on free days (hours)	8.14 (2.00)	0.056^**^	0.060^**^	0.038^*^
**Change from baseline (2020–2019)**				
ΔOvertime work (hours/month)	0.00 (45.05)	−0.026	−0.041^*^	−0.055^**^
ΔJob stressors	−0.30 (5.51)	−0.045^*^	−0.218^**^	−0.172^**^
ΔSocial support	0.18 (4.36)	0.003	−0.117^**^	−0.096^**^
ΔPsychological and physical stress response	−0.31 (11.02)	−0.040^*^	−0.417^**^	−0.193^**^
ΔPresenteeism (%)	−0.12 (4.22)	0.018	−0.147^**^	−0.470^**^
ΔPSQI global score	−0.06 (2.31)	−0.008	−0.145^**^	−0.101^**^
ΔTotal sleep time on workdays (hours)	0.14 (1.05)	0.118^**^	0.043^*^	0.052^**^
ΔTotal sleep time on free days (hours)	−0.11 (1.93)	−0.046^**^	−0.006	−0.005

*Job stressors, Social support, psychological, and physical stress response were measured by using the Brief Job Stress Questionnaire; higher scores indicate a less favorable job environment, less social support, or severer psychosomatic symptoms. Presenteeism was estimated by using the Work Limitation Questionnaire. Sleep disturbance was measured by using the Pittsburgh Sleep Quality Index; higher scores indicate poorer quality sleep. Significant at*

* *p <0.05*,

** *p <0.01*.

**Table 2:** Study participants: followed-up and dropped-out participants.

	**Followed-up participants**	**Missed participants**	**Comparison**
	***N* (%)**	***N* (%)**	
Total sample	3,552	966	
**Gender**			
Male	1,949 (54.9%)	423 (43.8%)	χ^2^ = 37.865^***^
Female	1,598 (45.0%)	542 (56.1%)	
	**Mean (SD)**	**Mean (SD)**	
Age (years)	37.4 (11.0)	35.8 (11.3)	*t* = 3.804^***^
**Job status (baseline: 2019)**			
Overtime work (hours/month)	21.2 (26.7)	17.2 (27.1)	*t* = 4.099^***^
Job stressors	39.7 (6.6)	40.8 (7.2)	*t* = −4.373^***^
Social support	19.5 (5.3)	20.0 (5.5)	*t* = −2.952^**^
**Outcomes (baseline: 2019)**			
Psychological and physical stress response	57.2 (13.7)	59.6 (15.5)	*t* = −4.789^***^
Presenteeism (productivity loss) (%)	5.9 (6.7)	6.6 (5.0)	*t* = −2.984^**^
**Sleep (baseline: 2019)**			
Sleep disturbance	6.44 (2.78)	6.75 (3.17)	*t* = −2.954^**^
C1: Sleep quality	1.43 (0.74)	1.47 (0.78)	*t* = −1.296
C2: Sleep latency	1.12 (0.99)	1.25 (1.01)	*t* = −3.492^***^
C3: Sleep duration	1.53 (0.82)	1.48 (0.86)	*t* = 1.497
C4: Habitual sleep efficiency	0.20 (0.55)	0.27 (0.68)	*t* = −3.333^**^
C5: Frequency of sleep disturbance	1.03 (0.53)	1.08 (0.55)	*t* = −2.372^*^
C6: Use of sleep medication	0.12 (0.51)	0.16 (0.61)	*t* = −2.282^*^
C7: Daytime dysfunction	1.02 (0.77)	1.05 (0.83)	*t* = −1.105
Total sleep time on workdays (hours)	6.12 (1.09)	6.19 (1.18)	*t* = −1.683
Total sleep time on free days (hours)	8.17 (1.97)	8.40 (1.85)	*t* = −3.237^**^

*Job stressors, Social support, psychological, and physical stress response were measured by using the Brief Job Stress Questionnaire; higher scores indicate a less favorable job environment, less social support, or severer psychosomatic symptoms. Presenteeism was estimated by using the Work Limitation Questionnaire. Sleep disturbance was measured by using the Pittsburgh Sleep Quality Index; higher scores indicate poorer quality sleep. Significant at*

* *p <0.05*,

** *p <0.01*,

*** *p <0.001*.

**Table 3:** Correlation and reliability scales.

	**Pearson's** ***r*** **correlation with (2020/2019)**					**Cronbach α (2020)**
	**1**	**2**	**3**	**4**	**5**	
1. Job stressors		0.616^**^	0.504^**^	0.405^**^	0.311^**^	0.786
2. Psychological and physical stress response	0.529^**^		0.420^**^	0.327^**^	0.234^**^	0.930
3. Social support	0.359^**^	0.332^**^		0.502^**^	0.292^**^	0.871
4. Sleep disturbance	0.268^**^	0.548^**^	0.247^**^		0.414^**^	0.735
5. Presenteeism	0.390^**^	0.571^**^	0.263^**^	0.405^**^		0.852
Cronbach α (2019)	0.779	0.929	0.876	0.733	0.858	

*Correlations for scales surveyed 2019 are shown above the main diagonal. Correlations for scales surveyed in 2020 are shown below the main diagonal. Crombach α for scales surveyed in 2019 are displayed in the horizontal axis, and for the year 2020 in the vertical axis. Job stressors, Social support, psychological, and physical stress response were measured by using the Brief Job Stress Questionnaire; higher scores indicate a less favorable job environment, less social support, or severer psychosomatic symptoms. Presenteeism was estimated by using the Work Limitation Questionnaire. Sleep disturbance was measured by using the Pittsburgh Sleep Quality Index; higher scores indicate poorer quality sleep. Significant at*

* *p <0.05*,

** *p <0.01*.

Table 4 compares the baseline values and the changes in the values of each group categorized by the frequency of remote work. There were significant differences at baseline in age (*F* = 29.60, *p* < 0.001), overtime work (*F* = 9.70, *p* < 0.001), job stressors (*F* = 46.85, *p* < 0.001), and total sleep time on free days (*F* = 3.65, *p* = 0.012). No difference was found at baseline for social support, total sleep time on workdays, and psychological and physical stress responses. Regarding 1 year changes, job stressors (*F* = 5.42, *p* = 0.001), total sleep time on workdays (*F* = 15.08, *p* < 0.001), total sleep time on free days (*F* = 2.784, *p* = 0.039), and psychological and physical stress responses (*F* = 4.249, *p* = 0.005) were identified as variables showing a significant difference between the two groups.

**Table 4:** Comparison of variables at baseline and their changes from 2019 to 2020.

	**Total**		**Remote work frequency (/week): mean (SD)**								***F*-value**
			**0 days** **(None)**		**1–2 days**		**3–4 days**		**5 days** **(“full-remote”)**		
**Baseline (2019)**	**Mean**	**(SD)**	**Mean**	**(SD)**	**Mean**	**(SD)**	**Mean**	**(SD)**	**Mean**	**(SD)**	
Age (years)	37.30	(10.86)	38.32	(11.87)	38.84	(10.97)	34.97	(8.49)	33.76	(8.47)	29.60^***^
Overtime work (hours/month)	22.21	(26.97)	24.27	(30.25)	22.40	(25.09)	20.34	(22.52)	15.08	(21.95)	9.7^***^
Job stressors	40.32	(6.67)	41.66	(6.48)	40.07	(6.34)	38.77	(6.69)	37.75	(6.88)	46.85^***^
Social support	19.58	(5.42)	19.74	(5.54)	19.57	(5.13)	19.13	(5.30)	19.99	(5.82)	2.54
Psychological and physical stress response	57.55	(13.70)	57.69	(13.49)	57.31	(13.96)	57.35	(13.73)	57.96	(14.04)	0.25
Presenteeism (%)	6.09	(4.53)	6.20	(4.64)	5.80	(4.32)	6.11	(4.39)	6.27	(4.81)	1.41
Sleep disturbance	6.45	(2.75)	6.42	(2.81)	6.55	(2.77)	6.37	(2.58)	6.60	(2.73)	0.87
Total sleep time on workdays (hours)	6.10	(1.11)	6.04	(1.14)	6.12	(1.12)	6.17	(1.06)	6.15	(1.12)	2.34
Total sleep time on free days (hours)	8.14	(2.00)	8.04	(2.11)	8.15	(1.82)	8.25	(1.94)	8.43	(1.94)	3.65^*^
**Change**											
ΔOvertime work (hours/month)	0.00	(45.05)	1.57	(60.44)	−0.77	(24.78)	−2.14	(24.45)	−0.66	(28.88)	1.22
ΔJob stressors	−0.30	(5.51)	0.03	(5.46)	−0.78	(5.35)	−0.22	(5.70)	−1.14	(5.53)	5.42^**^
ΔSocial support	0.18	(4.36)	0.19	(4.45)	0.07	(4.27)	0.24	(4.39)	0.22	(4.04)	0.22
ΔPsychological and physical stress response	−0.31	(11.02)	0.20	(11.05)	−1.03	(10.59)	−0.03	(11.49)	−2.06	(10.34)	4.25^**^
ΔPresenteeism (%)	−0.12	(4.22)	−0.16	(4.24)	−0.14	(4.07)	−0.12	(4.30)	0.23	(4.33)	0.60
ΔSleep disturbance	−0.06	(2.31)	−0.02	(2.25)	−0.16	(2.38)	−0.03	(2.33)	−0.12	(2.39)	0.73
ΔTotal sleep time on workdays (hours)	0.14	(1.05)	0.04	(1.11)	0.10	(0.93)	0.28	(1.03)	0.43	(1.05)	15.08^***^
ΔTotal sleep time on free days (hours)	−0.11	(1.93)	−0.05	(2.10)	−0.05	(1.73)	−0.20	(1.83)	−0.38	(1.78)	2.78

*Job stressors, Social support, psychological, and physical stress response were measured by using the Brief Job Stress Questionnaire; higher scores indicate a less favorable job environment, less social support, or severer psychosomatic symptoms. Presenteeism was estimated by using the Work Limitation Questionnaire. Sleep disturbance was measured by using the Pittsburgh Sleep Quality Index; higher scores indicate poorer quality sleep. Significant at*

* *p <0.05*,

** *p <0.01*,

*** *p <0.001 (ANOVA)*.

Table 5 shows the results of logistic regression analysis, setting the worsening of psychological and physical stress responses as the dependent variable. In model 1, in which only the frequency of remote work was included in the logistic regression, a tendency of improvement in mental health was observed, but the statistical significance was ambiguous [odds ratio (OR) = 0.654–0.950, *p* = 0.003–0.571]. In model 2, in which demographic variables, baseline status of job environment, and sleep was added to the analysis, the frequency of remote work had a significant negative association with the worsening of psychological and physical stress responses [adjusted ORs (aORs) = 0.525–0.803, *p* = <0.001–0.021]. In model 3, in which 1 year changes in the variables were added, the statistical significance of the baseline factors disappeared, and 1 year changes in the variables became statistically significant. Finally, in model 4, the statistically significant variables identified in model 3 were included in the analysis with the frequency of remote work to control for confounding factors, and indicated that remote work significantly associated with decreasing of psychological and physical stress response (1–2 days/week: aOR = 0.782, *p* = 0.003. 3–4 days/week: aOR = 0.833, *p* = 0.030; 5 days/week: aOR = 0.611, *p* < 0.001), with adjustment of the increase in job stressors (aOR = 1.160/points, *p* < 0.001), reduction of social support (aOR = 1.068/pt, *p* < 0.001), worsening of sleep disturbance (PSQI) (aOR = 1.268/pt, *p* < 0.001), and increased total sleep time on workdays (aOR = 1.179/h, *p* < 0.001).

**Table 5:** Logistic regression analysis of risk factors for worsening psychological and physical stress responses.

**Independent variables**	**Model 1**		**Model 2**		**Model 3**		**Model 4**				
	**OR**	***p***	**aOR**	***p***	**aOR**	***p***	**aOR**	**95% CI**			***p***
**Remote work**											
1–2 days/week	0.783	0.008	0.737	0.001	0.869	0.178	0.782	0.662	–	0.922	0.003
3–4 days/week	0.950	0.571	0.803	0.021	0.916	0.409	0.833	0.707	–	0.982	0.030
5 days/week (“full-remote”)	0.654	0.003	0.525	<0.001	0.681	0.021	0.611	0.456	–	0.819	<0.001
Gender: (0 = Female, 1 = male)			1.031	0.700	1.041	0.642					
Age			0.989	0.003	0.994	0.163					
Baseline overtime work (hours/month)			0.997	0.027	0.997	0.060			
Baseline job stressors			0.963	<0.001	1.011	0.150			
Baseline social support			0.982	0.015	0.989	0.239			
Baseline sleep disturbance			0.953	0.004	0.997	0.881			
Baseline total sleep time on workdays (hours)			0.977	0.564	0.992	0.877			
Baseline total sleep time on free days (hours)			0.976	0.214	0.997	0.912			
ΔOvertime work (hours/month)					1.000	0.746			
ΔJob stressors					1.164	<0.001	1.160	1.139	–	1.181	<0.001
ΔSocial support					1.063	<0.001	1.068	1.047	–	1.089	<0.001
ΔSleep disturbance					1.265	<0.001	1.268	1.215	–	1.323	<0.001
ΔTotal sleep time on workdays (hours)					1.167	0.003	1.179	1.079	–	1.289	<0.001
ΔTotal sleep time on free days (hours)					1.036	0.179					

*Job stressors, Social support, psychological, and physical stress response were measured by using the Brief Job Stress Questionnaire; higher scores indicate a less favorable job environment, less social support, or severer psychosomatic symptoms. Presenteeism was estimated by using the Work Limitation Questionnaire. Sleep disturbance was measured by using the Pittsburgh Sleep Quality Index; higher scores indicate poorer quality sleep. OR, Odds ratio; aOR, adjusted OR; 95%CI, 95% bootstrap Confidence Interval*.

Table 6 shows the results of logistic regression analysis, in which worsening presenteeism was set as the dependent variable. When putting only the frequency of remote work (model 1) and adding the demographic variables (model 2), the baseline status of job environment, psychological and physical stress responses, and sleep to the analysis, there was almost no significant difference between the two models. In model 3, when 1 year change was added as a variable, remote work of 5 days, changing job stressors, social support, psychological and physical stress responses, and sleep disturbance were found to be significant factors for worsening presenteeism. Finally, in model 4, the significant variables detected in model 3 were put into the analysis with the frequency of remote work to control for confounding factors, and shown that 5 days a week of remote work (full-remote) was a significant factor for worsening presenteeism (aOR = 1.421, *p* = 0.017) with the adjustment of increasing job stressors (aOR = 1.036/pt, *p* < 0.001), reduction of social support (aOR = 1.033/pt, *p* < 0.001), worsening of psychological and physical stress responses (aOR = 1.049/pt, *p* < 0.001), and worsening of sleep disturbance (PSQI) (aOR = 1.080/pt, *p* < 0.001).

**Table 6:** Logistic regression analysis of risk factors for worsening presenteeism.

**Independent variables**	**Model 1**		**Model 2**		**Model 3**		**Model 4**				
	**OR**	***p***	**OR**	***p***	**OR**	***p***	**OR**	**95%CI**			***p***
**Remote work**											
1–2 days/week	0.914	0.329	0.892	0.219	1.008	0.937	0.999	0.825	–	1.209	0.990
3–4 days/week	1.053	0.570	0.989	0.908	1.092	0.388	1.076	0.890	–	1.301	0.452
5 days/week (“full-remote”)	1.189	0.214	1.092	0.542	1.422	0.024	1.421	1.064	–	1.896	0.017
Gender: (0 = Female, 1 = male)			1.078	0.337	1.160	0.079					
Age			0.993	0.052	0.999	0.895					
Baseline overtime work (hours/month)			0.999	0.673	1.001	0.712			
Baseline job stressors			0.990	0.160	1.004	0.633			
Baseline social support			0.994	0.425	1.004	0.686			
Baseline psychological and physical stress responses			0.992	0.043	1.009	0.058			
Baseline sleep disturbance			1.024	0.204	1.024	0.282			
Baseline total sleep time on workdays (hours)			1.026	0.526	1.005	0.922			
Baseline total sleep time on free days (hours)			1.008	0.685	1.035	0.171			
ΔOvertime work (hours/month)					1.001	0.250			
ΔJob stressors					1.038	<0.001	1.036	1.019	–	1.053	<0.001
ΔSocial support					1.033	0.002	1.033	1.014	–	1.053	<0.001
ΔPsychological and physical stress responses					1.053	<0.001	1.049	1.039	–	1.058	<0.001
ΔSleep disturbance					1.106	<0.001	1.080	1.042	–	1.118	<0.001
ΔTotal sleep time on workdays (hours)					1.048	0.340					
ΔTotal sleep time on free days (hours)					1.042	0.102					

*Job stressors, Social support, psychological, and physical stress response were measured by using the Brief Job Stress Questionnaire; higher scores indicate a less favorable job environment, less social support, or severer psychosomatic symptoms. Presenteeism was estimated by using the Work Limitation Questionnaire. Sleep disturbance was measured by using the Pittsburgh Sleep Quality Index; higher scores indicate poorer quality sleep. OR, Odds ratio; aOR, adjusted OR; 95%CI, 95% bootstrap Confidence Interval*.

## Discussion

This empirical study provides evidences that remote work decreases psychological and physical stress responses when controlling the confounding factors such as for job stressors, social support, and sleep status as personal intervening factors. On the other hand, the effects of remote work on presenteeism were limited, although full-remote work was found to have a negative effect on presenteeism.

Although information technology, which assists remote work has remarkably advanced in recent years and it is slightly hard to apply in this circumstance directly, there are some previous reports in the literature that assessed the effects of remote work on mental health, work productivity, and presenteeism, but the conclusions were inconsistent (Di Martino and Wirth, 1990; Bailey and Kurland, 2002; Martin and MacDonnell, 2012; Baert et al., 2020; Steidelmüller et al., 2020). This inconsistency may be a result of the lack of consideration of confounding factors. As remote work is just one of the factors affecting workers' mental health and productivity, the effects of job stressors, the surrounding environment, and personal factors, such as sleep, should be adjusted when discussing the effects of remote work on workers' mental health and productivity (Furuichi et al., 2020). Moreover, these factors, particularly support or conflict within the family, play important roles in how well a worker adapts to remote work (Darouei and Pluut, 2021). For example, working while taking care of children, working in a noisy home environment, or loneliness during remote work may affect stress reactions and work productivity.

The results of the present study showed a weak and unstable statistical significance before adjusting for these factors, indicating the importance of controlling them, and suggested strategies to reduce stress responses and to improve work productivity of remote work. Independently from remote work status, an increase in job stressors, decrease in social support, and worsening of sleep were risk factors of worsening stress reactions and loss of work productivity.

Higher productivity and less stress reactions while performing remote work may be possible by improving job environments, such as quantitative/qualitative job load, physical demands, job control, skill utilization, interpersonal conflict, physical environment, job suitability, and meaningfulness of work. Furthermore, maintaining and promoting social support between workers and their supervisors, colleagues, family, and friends, and sleeping well, which will be possible by improving sleep hygiene (Stepanski and Wyatt, 2003; Shimura et al., 2020), such as avoiding night-cap, avoiding the use of electronic devices in bed, exposing oneself to sunlight in the morning, keeping to regular mealtimes, and eating a sufficient amount of vegetables are also important. Some of these factors are the responsibility of the companies, and some must be done by the workers themselves as a self-care.

As a measure against COVID-19, keeping a social distance is a public health requirement, and improving workers' mental health is also simultaneously required (Fingret, 2000). Remote work could be a useful tool to balance them, although there are few studies to date assessing effective methods for improving occupational mental health (Richardson and Rothstein, 2008). More than one-third of firms that had employees switch to remote work during the COVID-19 pandemic believe that remote work will remain more common at their company even after the pandemic ends (Bartik et al., 2020).

The results of this study are thought to help organizations in deciding whether to continue remote work or not. Meanwhile, the exact mechanism and the path between remote work and psychological and physical stress responses and presenteeism were not clarified in this study. There are various hypotheses and factors involved in this association, such as being able to work in a relaxing room environment, not being distracted by the gaze of surrounding people in the office, no need to commute, and so on (Bailey and Kurland, 2002; Martin and MacDonnell, 2012). Whereas, partial-remote work did not affect work productivity, full-remote work was shown to reduce work performance. There is a possibility that workers with illnesses or in poor condition, such as with a cold or any severe health disfunctions, are unable to go to work but can still keep working remotely, and this may apparently worsen presenteeism.

As a limitation, firstly, this study was a survey of only tertiary industries in a limited regional area. Therefore, generalization of the results should be performed with caution. Secondly, this was an observational study of only 2 years. A follow-up study investigating the effects of switching back to normal work from remote work, or the intervention studies, such as randomized controlled trials, are needed in the future to analyze the exact effects of remote work on workers' mental health and presenteeism. Thirdly, as mentioned above, other factors that substantially affect stress reactions and presenteeism, such as having opportunities to relax, a noisy home environment, not being distracted by the gaze of surrounding people in the office, having to care for young children, or commuting time and method, were not assessed in this study, and should hence be analyzed in a future study. Fourthly, we could not follow up all of the participants who initially joined the study in 2019. The drop-out rate was 21.4%, which is higher than the average annual job retirement rate of about 15% (Male 13%, Female 17%) in Japan (Ministry of Health Labour, and Welfare, 2020). This may be a result of survivorship bias, in which workers who could not adapt well to remote work might have dropped out of the study.

## Conclusion

Remote work can reduce psychological and physical stress responses. The effects of remote work on presenteeism is limited, although full-remote work can result in presenteeism. From the viewpoint of occupational mental health, the review of working styles is expected to be beneficial, even after the end of the COVID-19 pandemic.

## Data Availability Statement

The raw data supporting the conclusions of this article will be made available by the authors, without undue reservation.

## Ethics Statement

The studies involving human participants were reviewed and approved by Tokyo Medical University Medical Ethics Review Board. The patients/participants provided their written informed consent to participate in this study.

## Author Contributions

AS: conceptualization, data curation, formal analysis, funding acquisition, investigation, methodology, project administration, and writing—original draft. KY: formal analysis, data curation, and writing—original draft. YI: validation, formal analysis, and writing—review and editing. YA: conceptualization, resources, data curation, and writing—review and editing. TI: conceptualization, validation, investigation, writing—review and editing, and supervision. All authors contributed to the article and approved the submitted version.

## Funding

This work was supported by JSPS KAKENHI Grant No. JP20K07955.

## Conflict of Interest

AS has received lectures fees from Dainippon Sumitomo Pharma, MSD, and Eisai outside of the submitted work. AS and YA are board members of Children & Future Co., Ltd. KY and YI are partially employed by Children & Future Co., Ltd, and have received personal fees outside of the submitted work. TI has received personal fees from Mochida Pharmaceutical, Takeda Pharmaceutical, Eli Lilly, Janssen Pharmaceutical, MSD, Taisho Toyama Pharmaceutical, Yoshitomiyakuhin, Ono Pharmaceutical, Otsuka Pharmaceutical, Dainippon Sumitomo Pharma, Mitsubishi Tanabe Pharma, Kyowa Pharmaceutical Industry, Pfizer, Shionogi, Tsumura, Novartis Pharma, Eisai, Daiichi Sankyo and Meiji Seika Pharma outside of the submitted work; and is a member of the advisory boards of Pfizer, Novartis Pharma, and Mitsubishi Tanabe Pharma.

## Publisher's Note

All claims expressed in this article are solely those of the authors and do not necessarily represent those of their affiliated organizations, or those of the publisher, the editors and the reviewers. Any product that may be evaluated in this article, or claim that may be made by its manufacturer, is not guaranteed or endorsed by the publisher.
